# Alternations of Metabolic Profiles in Synovial Fluids and the Correlation with T2 Relaxation Times of Cartilage and Meniscus—A Study on Anterior Cruciate Ligament- (ACL-) Injured Rabbit Knees at Early Stage

**DOI:** 10.1155/2019/8491301

**Published:** 2019-07-30

**Authors:** Hongyue Tao, Yiwen Hu, Yang Qiao, Yuxue Xie, Tianwu Chen, Shuang Chen

**Affiliations:** ^1^Department of Radiology and Institute of Medical Functional and Molecular Imaging, Huashan Hospital, Fudan University, Shanghai, China; ^2^Department of Sports Medicine, Huashan Hospital, Fudan University, Shanghai, China

## Abstract

**Objectives:**

To examine the metabolic profiles alterations of synovial fluids from anterior cruciate ligament- (ACL-) injured rabbit knees at early stage and analyze the correlation with T2 relaxation times of cartilage and meniscus.

**Methods:**

The right knees of 15 rabbits were selected for the construction of ACL injury models, whereas the contralateral knees served as control group. After 4 weeks, both knees were examined by MRI with quantitative T2 mapping sequence, and the T2 relaxation times of cartilage and meniscus were measured. Then, the synovial fluids were obtained from both knee capsules and performed liquid chromatography-mass spectrometry analysis (LC-MS).

**Results:**

The T2 relaxation times of cartilage and meniscus in ACL-injured knees were significantly higher than those in control knees (Cartilage: 41.52 ± 2.98 ms vs 36.02 ± 2.71 ms,* P *< 0.001; Meniscus: 33.35 ± 3.57 ms vs 27.27 ± 2.10 ms,* P *< 0.001). Twenty-eight differential metabolites were identified based on a total of 1569 detected signatures between ACL-injured knees and control knees. These differential metabolites primarily implied perturbations in the fluxes of lipids and steroid-based compounds. The Linear regression analysis demonstrated satisfactory correlations between glycerophospholipid metabolism and T2 relaxation times of both cartilage and meniscus in ACL-injured knees (R^2^ = 0.8204 and 0.8197, respectively).

**Conclusion:**

ACL injury of rabbit knees resulted in elevated T2 relaxation times of cartilage and meniscus and perturbed metabolism of various lipids and steroids in synovial fluids, particularly glycerophospholipids. Glycerophospholipid metabolism related compounds could serve as potential biomarkers for early degenerative changes of cartilage and meniscus after ACL injury.

## 1. Introduction

Anterior cruciate ligament (ACL) tear is one of the most common forms of knee injury and poses a significant risk of permanent disability to the afflicted knee [1]. ACL injury has been linked to the development of posttraumatic osteoarthritis, particularly in younger patients, due to its contribution to recurrent knee instability [2, 3]. Degeneration and damage of cartilage and meniscus are the main pathophysiological features of osteoarthritis. Currently, MRI T2-mapping has previously been shown to be a powerful tool for assessing biochemical changes in the cartilage and meniscus prior to the occurrence of morphological deterioration [4–7]. There is evidence that elevated T2 relaxation times could be associated with the disorganization of collagen and increased water content in the cartilage and meniscal matrices, which could be indicators of early degeneration in these regions [7, 8].

Metabolite profiling has shown great utility in helping researchers identify biomarkers and understand the pathogenesis of complex diseases [9]. One of the inherent strengths of metabolomics lies in the fact that the metabolome, consisting of all small molecule chemicals present within a biological system, is comparatively more sensitive and less complex than the genome, transcriptome, or proteome [10]. Metabolites exhibit the most rapid response to various physiological and environmental stimuli, thereby presenting the most current snapshot of the biological system under investigation. In particular, recent advances in mass spectrometric technologies have enabled real-time metabolic profiling that shows remarkable sensitivity and accuracy. All these advantages have made metabolomics an attractive analytic strategy for the elucidation of disease mechanisms and the development of novel, more accurate diagnostic methods. Synovial fluid contains a complex mixture of biochemical components that help nourish and lubricate cartilage and meniscus. Thus, metabolic profiling of synovial fluid could provide important insights into joint-related pathophysiological alterations [11, 12].

In fact, there is already some evidence that associates certain synovial fluid constituents, such as amino acids, choline, and creatine, with osteoarthritis or rheumatoid arthritis [13–16]. These results prompted us to speculate that the metabolic profile of synovial fluid could also provide important information on early stage of posttraumatic osteoarthritis after ACL injury. Therefore, the aim of this study is to investigate the validity of this hypothesis by characterizing the metabolic changes that occur in synovial fluid following the onset of ACL injury-induced osteoarthritis and evaluating whether such alterations correlate with pathological remodeling in the cartilage and meniscus captured by MRI T2 mapping.

## 2. Materials and Methods

### 2.1. Construction of Animal Model

All animal experiments were approved by the Ethical Committee Review Board of our Animal Department. The initial study cohort consisted of 18 healthy male white New Zealand rabbits aged 3–4 months and weighing 2.5–3.0kg. The right knee of each rabbit was selected for constructing the ACL injury model. Briefly, the rabbits were anesthetized by intravenously administrating 3% pentobarbital at a dose of 30mg/kg body weight and placed supine on the operating table. The right knee was approached through medial parapatellar incision with the patella dislocated laterally. The native ACL was exposed and cut in the middle. The left knees, as controls, were similarly incised and dislodged without cutting the ACL. Once the operation was completed, the patella was restored and the joint capsule was closed with interrupted sutures, followed by the closure of the wound in anatomical layers. No animal death occurred during or following the surgery. All rabbits were subsequently allowed to move freely in their cages and received a daily injection of 80,000 U penicillin for 3 days. We performed MRI imaging and metabolomics studies 4 weeks after the surgery, based on the previous evidence that this is when surgically induced inflammatory reaction would diminish and early degenerative changes would occur in the cartilage [17]. Three rabbits with slightly infected wound were excluded. As a result, 15 rabbits were used as test subjects.

### 2.2. MRI Examination and Acquisition of Synovial Fluid Samples

Anesthesia was induced by intravenous administration of 3% pentobarbital. MRI was performed on both knees of each rabbit using a 3.0 T MRI scanner (Siemens Verio, Germany) with a gradient strength of 40 mT/m and a single-channel surface coil. Scan sequences included fat-saturated proton density-weighted (FS-PD) imaging and T2 mapping. The FS-PD sequence was performed with the following settings: repetition time (TR) 6000 ms; echo time (TE) 89 ms; flip angle 150°; field of view (FOV) 600 × 600 mm; image matrix 250 × 250; slice thickness 1.1 mm; number of excitation 2; total scan time 5 min and 30 sec. T2 mapping was performed with multi-echo spin-echo pulse sequences, using the following settings: TR 1700 ms; TE 12, 24, 36, 48, and 60 ms; flip angle of refocusing pulse 180°, flip angle of excitation pulse 90°; FOV 120 × 120 mm; image matrix 250 × 250; slice thickness 1.1 mm; number of excitation 2; total scan time 21 min and 10 sec. Images were acquired in the sagittal plane of each knee. Four cartilage compartments, including the medial femoral condyle, lateral femoral condyle, medial tibial plateau, and lateral tibial plateau, as well as four meniscal compartments, including the anteromedial, posteromedial, anterolateral, and posterolateral horns, were identified. The T2 relaxation time of each abovementioned compartment was measured by T2 mappings generated from pixel-wise, mono-exponential nonnegative least square fit analyses. The T2 relaxation time of the entire cartilage was calculated as the average of all its compartments. The T2 relaxation time of the meniscus was obtained according to the same method.

Immediately following the MRI examinations, the rabbits were maintained under anesthesia and their knees were disinfected by 75% ethanol. Subsequently, 0.5 mL of physiological saline was injected into each knee capsule via the medial approach by using a 1-mL sterile syringe. The syringe was left in the capsule for 1 min and then used to withdraw 0.25 mL of synovial fluid, which was immediately transferred to a sterile microcentrifuge tube and frozen in liquid nitrogen.

### 2.3. Detection and Identification of Synovial Fluid Metabolites via LC-MS

Separation and detection of synovial fluid metabolites were performed on a 6530 Accurate-Mass Q-TOF LC/MS System with 1290 Infinity LC (Agilent, USA). Briefly, 4 *μ*L of the synovial fluid sample was directly loaded onto a Zorbax C18-column (100 × 2.1 mm, 1.8 *μ*m, Agilent, USA) maintained at 40°C. The column was then washed with a mobile phase of 0.1% (v/v) formic acid in an acetonitrile gradient at a constant flow rate of 0.4 mL/min. The gradient of acetonitrile was first set to 5% (v/v, in water) for 2 min and then linearly increased to 95% during the next 11 min before being maintained at this level for another 2 min.

MS spectra were acquired under negative-ion mode with the following instrument settings: mass-to-charge (m/z) range 50–1000, source temperature 100°C, nebulizing gas: nitrogen, desolvation temperature 35°C; desolvation gas flow 600 L/h, capillary voltage 4 kV, cone gas: nitrogen, cone gas flow 50 L/h, cone voltage 35 kV, extraction voltage 4 V, scan time 0.03s, and interscan delay – 0.02 s.

Instrument calibration was performed by using a standard leucine-enkephalin sample as a lock-mass, which would generate an [M-H]- ion of 554.2615Da under negative-ion mode. A series of identical LC-MS experiments were conducted with the standard and coefficient of variation (CV) analysis was used to evaluate the reproducibility of the analytic method.

### 2.4. Data Analysis

Statistical analysis was performed using SPSS (version 22.0). The T2 relaxation times of cartilage and meniscus were expressed as means ± standard deviation. Comparisons of two groups were conducted using paired t-test since the MRI data of two groups were accorded with normal distribution.* P *< 0.05 was considered statistically significant

The raw LC-MS data was autoscaled and mean-centered with XCMSonline to minimize the potential impact of unit differences. Samples were then normalized according to the total area and subjected to multivariate analysis using SIMCA-p software (version 12.0, Umetrics AB, Sweden). Specifically, global differences between the metabolic profiles of ACL-injured knees and controls were identified and analyzed by principle component analysis (PCA), partial least squares discriminant analysis (PLS-DA), and orthogonal partial least squares discriminant analysis (OPLS-DA). Cross-validation of PLS–DA results was conducted with R software. The PLS-DA model was considered acceptable if its Q2 value was above 40% [18]. The variable importance in the projection (VIP) score and* P* value of each metabolite were calculated from the validated OPLS-DA model and t-tests, respectively. Metabolites with VIP > 1 and* P*<0.001 were chosen for further functional analysis.

The MS data of each selected metabolite was queried against the METLIN database for chemical identification. Pathway analysis was performed with MetaboAnalyst 3.0 to identify biological patterns that had been altered in the ACL injury group.

Differential metabolites were selected for model construction based on the low* P* value and the high impact score of the pathway. Linear regression analysis was used to determine the mathematical relationship between the levels of key differential metabolites and the T2 relaxation times of the cartilage and meniscus. Coefficients of determination (R^2^) were calculated by Pearson correlation analysis.

## 3. Results

### 3.1. MRI Assessment of Cartilage and Meniscus

**Figure 1:**
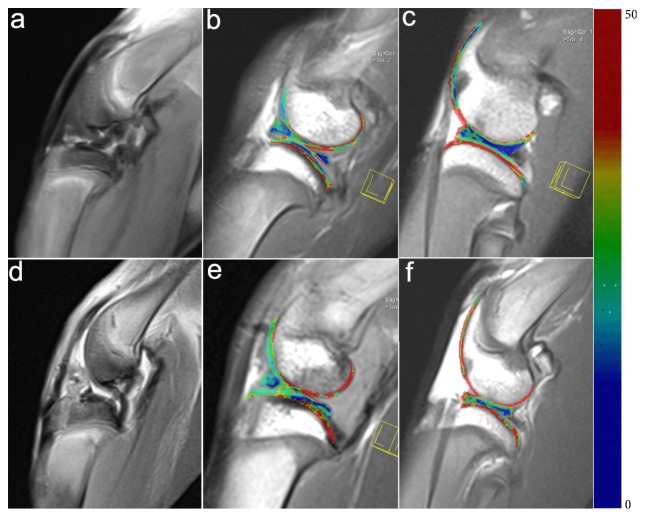
MRI scans of control and ACL-injured rabbit knees. Descriptions of the images are as follows: Sagittal image acquired from fat-saturated proton density-weighted (FS-PD) (a) as well as T2 mappings of the medial (b) and lateral (c) tibiofemoral joint in the control knee; sagittal image of PD-FS (d) and T2 mappings of the medial (e) and lateral (f) tibiofemoral joint in the ACL-injured knee. The ACL-injured knees showed clear signs of ACL tear (a) compared to the contralateral control (d). On T2 mapping, according to the color bar on the right, the T2 relaxation times of the cartilage and meniscus in the ACL-injured knee were significantly higher than those of the control knee.

The right knees of the rabbits all showed clear signs of ACL tear compared to the contralateral knees, confirming successful model construction. In ACL-injury knees, the T2 relaxation times of the four cartilage compartments were medial femoral condyle 44.15 ± 4.23 ms, lateral femoral condyle 44.03 ± 4.28 ms, medial tibial plateau 37.82 ± 7.07 ms, and lateral tibial plateau 40.44 ± 8.32 ms. On the other hand, the T2 relaxation times of the meniscal compartments were anteromedial 33.98 ± 6.75 ms, posteromedial 33.85 ± 5.84 ms, anterolateral 34.93 ± 7.66 ms, and posterolateral 30.73 ± 6.72 ms. For the contralateral knees, the T2 relaxation times of the cartilage compartments were medial femoral condyle 38.92 ± 3.45 ms, lateral femoral condyle 37.17 ± 3.45 ms, medial tibial plateau 33.01 ± 5.36 ms, and lateral tibial plateau 34.82 ± 4.49 ms. Meanwhile, the T2 relaxation times of the meniscal compartments were anteromedial 27.31 ± 3.67 ms, posteromedial 28.62 ± 2.79, anterolateral 27.54 ± 3.86 ms, and posterolateral 25.54 ± 3.10 ms. For each group, we calculated the mean T2 relaxation time of the cartilage by averaging those of the four cartilage compartments. We then similarly obtained the mean T2 relaxation time of the menisci. The mean T2 relaxation times of the cartilage and menisci in the ACL-injured knees were significantly higher than in the control knees (cartilage: 41.52 ± 2.98 ms vs 36.02 ± 2.71 ms,* P *< 0.001; menisci: 33.35 ± 3.57 ms vs 27.27 ± 2.10 ms,* P *< 0.001). This suggested that degenerative changes had occurred in both the cartilage and menisci of the ACL-injured knees. (Figure 1)

### 3.2. Metabolic Profiling of Synovial Fluids

**Figure 2:**
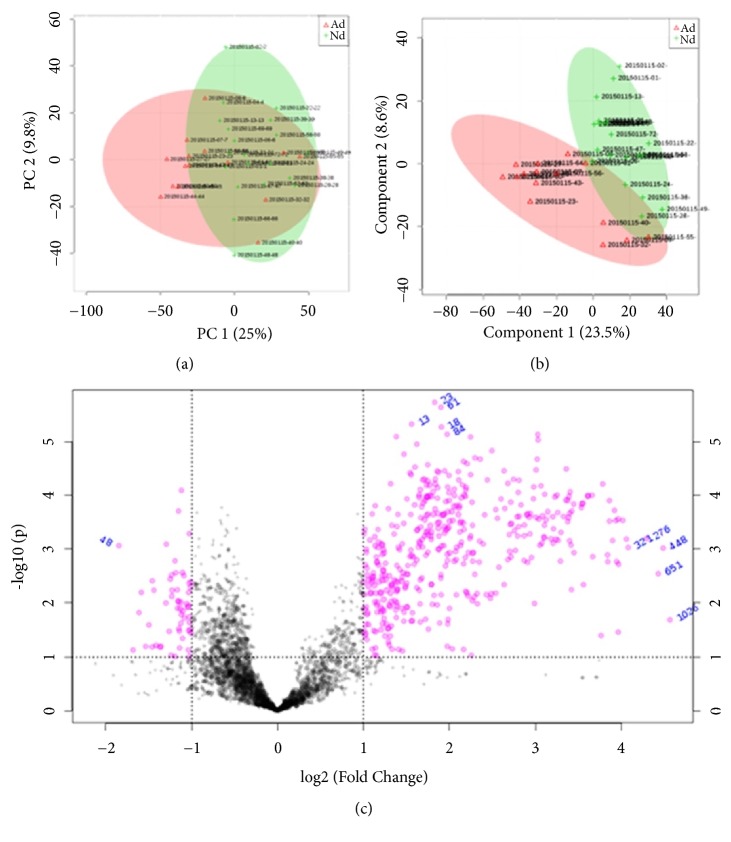
Metabolomic analysis and identification of differential metabolites. PCA and PLS-DA plots indicating clear differences in metabolic profiles between the ACL injury group (Ad, red triangle and red ellipse) and the control group (Nd, green diamond and green ellipse) (a, b). A volcano plot showing the distribution of all and differential metabolites based on their fold-change values (x-axis, on a logarithmic scale) and p-values (y-axis, on a logarithmic scale) (c).

Untargeted LC-MS profiling of synovial fluids collected from both knees led to the detection of 1569 distinct metabolite features. Data reproducibility of the detection method was evaluated by running a standard lock-mass and the average CV was calculated to be 10.9%, suggesting that the measurements were highly consistent. The PCA plot indicated a clear separation between the ACL injury group and the control group (Figure 2(a)). Moreover, 90% of all detected metabolites showed a CV value below 20%, indicating negligible random errors and good data reproducibility. The PLS-DA plot further confirmed the unambiguous separation between the two groups (Figure 2(b)). The Q2 of the PLS-DA model was calculated to be 46.29%, which exceeded the threshold value of 40% required for model validity [18].

**Figure 3:**
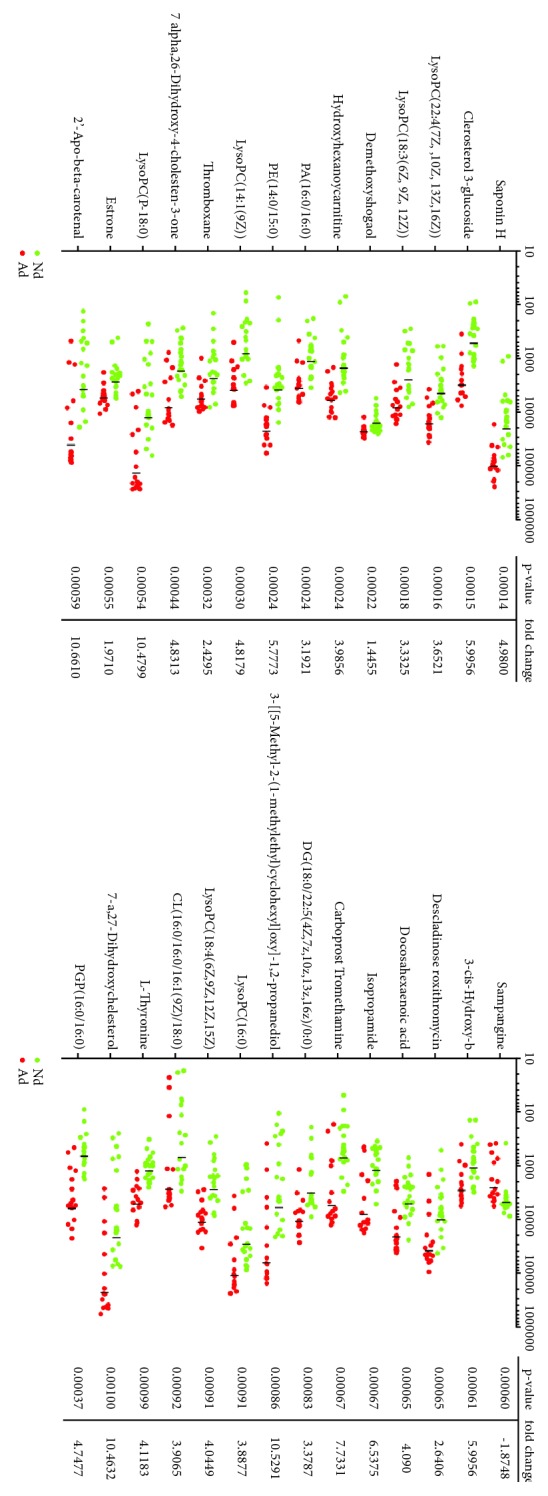
Summary of all differential metabolites related to glycerophospholipid metabolism and their respective fold-change values as calculated by XCMSonline and SPSS. Each green and red dot represent a control knee and a ACL-injured knee, respectively. The average fold-change of each metabolite in the ACL injury group or the control group is denoted by a vertical line.

We calculated the VIP score and P value of each identified metabolite (Figure 2(c)). Based on VIP > 1 and* P *< 0.001, we identified 28 differential metabolites, which were chemically identified and summarized in Figure 3 with their respective fold-change values. As shown, the overwhelming majority of these metabolites were significantly upregulated in the ACL injury group, with candidates such as lyso-PC (P-18:0), 2'-apo-beta-carotenal, 3-[[5-methyl-2-(1-methylethyl)cyclohexyl]oxy]-1,2-propanediol, and 7-a,27-dihydroxycholesterol exhibiting as much as 10-fold increase in concentration among the knees with ACL injury compared to the controls.

The biological roles of the differential metabolites were explored by MetaboAnalyst 3.0. As summarized in Table 1, ACL injury-related pathways included the metabolism of various glycerophospholipids, glycerolipids, and arachidonic acids, as well as the biosynthesis of primary bile acid, glycosylphosphatidylinositol anchors, unsaturated fatty acids, and steroid hormones. Overall, these results implied significant perturbations in the fluxes of lipids and steroid-based compounds. We subsequently focused on glycerophospholipid metabolism due to its low p value, which indicated strong statistical significance and its high impact score. Three differential metabolites, including lysophosphatidylcholine (lysoPC, 18:3(6Z, 9Z, 12Z)), phosphatidic acid (PA, 16:0/16:0), and phosphatidylethanolamine (PE, 14:0/15:0), were related to glycerophospholipid metabolism and thus selected for further correlation analysis.

### 3.3. Model Construction and Correlation Analysis

We constructed a mathematical model based on the levels of lysoPC(18:3(6Z, 9Z, 12Z)), PA(16:0/16:0), and PE(14:0/15:0) to predict the T2 relaxation times of the cartilage and meniscus in the injured knees. Linear regression analysis demonstrated that the mathematical relationships between the levels of the three metabolites and the T2 relaxation times could be represented by the equations below:

(1) log10⁡T2car=−0.0707×log10⁡A+1.8353

(2) log10⁡T2men=−0.0944×log10⁡A+1.8101

where A = (LysoPC(18:3(6Z, 9Z, 12Z) + PA(16:0/16:0) + PE (14:0/15:0))/3; T2_car_ and T2_men_ denote the T2 values in the knee cartilage and meniscus, respectively.

**Figure 4:**
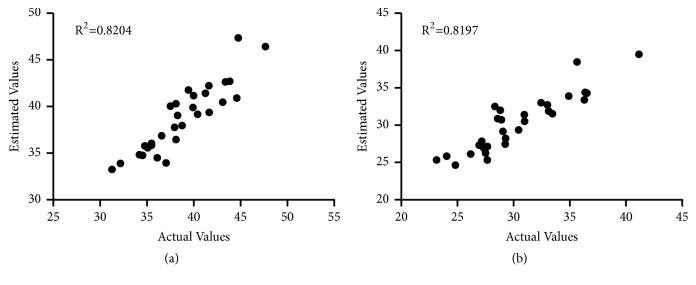
Linear regression analysis showing the correlation between the T2 values estimated from the predictive model and those measured from MRI T2 mapping for cartilage (a) and meniscus (b).

As illustrated in Figure 4, Pearson correlation analysis showed a coefficient of determination (R^2^) of 0.8204 for the first equation and 0.8197 for the second equation, which suggested that alterations of glycerophospholipid metabolism in synovial fluid were correlated with elevated T2 relaxation times of the cartilage and meniscus at the early stage after ACL injury.

## 4. Discussion

The main findings of the study were as follows: (1) We identified 28 metabolites that exhibited differential levels between ACL-injured knees and contralateral knees. (2) These differential metabolites primarily implied perturbations in the fluxes of lipids and steroid-based compounds. (3) The linear regression analysis demonstrated satisfactory correlations between glycerophospholipid metabolism and T2 relaxation times of both cartilage and meniscus in ACL-injured knees. Our results could facilitate the mechanistic elucidation of the onset of posttraumatic osteoarthritis after ACL injury.

Metabolomics analysis has been increasingly employed in orthopedic research. Mickiewicz et al. studied the metabolic profiles in synovial fluids obtained from knee joints of an ovine model [3]. The authors demonstrated that it was possible to distinguish between ACL surgical samples after 2 weeks and nonsurgical ones, based on their different metabolic signatures [3]. In another study, June et al. induced noninvasive ACL injury in mice by applying a mechanical load to their ankles and investigated the resultant metabolic alterations in microdissected joint tissues following ACL injury by HPLC-MS. Their results showed that the ACL injury caused a broad range of metabolic changes, including the upregulation of retinoid metabolism, phospholipid biosynthesis, hydroxyproline degradation, and anandamide metabolism [19]. Similar metabolomics studies have also been performed on the synovial fluid samples obtained from patients with osteoarthritis or rheumatoid arthritis [20, 21].

Among ACL injury-related pathways, we had discovered that glycerophospholipid metabolism had the most significant impact, which was corroborated by other similar studies. Xu et al. [22] performed metabolomic profiling on extracts prepared from osteophyte cartilage tissues and healthy controls, which found an array of chemical compounds with differential levels between the two groups, including amino acids, fatty acids, sulfonic acids, and glycerophospholipids. Another study was performed by Kosinska et al., where levels of different phospholipids could be used to differentiate between early osteoarthritis, late osteoarthritis, and rheumatoid arthritis [23]. As essential components of bilipid membranes, glycerophospholipids are known to play a critical role in joint protection. For example, Rahamim el al. reported that the polar lipids of synovial fluid in the temporomandibular joint of healthy individuals consisted primarily of phosphatidylcholine and other phospholipids, suggesting their involvement in disc lubrication [24]. Phospholipids have also been shown to act synergistically with hyaluronan to create boundary layers that serve as potent joint lubricants [25]. On the other hand, phospholipids can protect cellular components from reactive oxygen species, which have been shown to contribute to cartilage degeneration by inducing the depolymerization of hyaluronic acid [26] and promoting inflammation that ultimately leads to the secretion of extracellular matrix (ECM) degrading proteases [27, 28].

Glycerophospholipids have also been shown to be closely involved in estrogen-mediated maintenance of various joint tissues [29, 30]. A recent investigation conducted by Liu et al. of the metabolic perturbations caused by the administration of estradiol in osteoporosis patients revealed the central role of glycerophospholipid metabolism, which was further verified by changes in the activity level of phosphatidylcholine-sterol acyltransferase and in the concentration of lipid peroxidative products [31]. As estradiol and analogs have been shown to exert an inhibitory effect on cartilage degeneration and osteoclast activity [32], it is plausible that shifts in glycerophospholipid metabolism could play a mediating role in this process. Prostaglandins are well-established regulators of bone formation and resorption [33]. There is evidence that estrogen-type compounds such as 17-beta-estradiol could promote the in vivo production of several prostaglandins in the context of mechanical stress [34]. The role of glycerophospholipid metabolism in prostaglandin biosynthesis is likely related to the need to acquire arachidonic acids from various diacylglycerols and phospholipids via the action of different types of phospholipase [35, 36]. Prostaglandins are also known to play important proinflammatory roles and upregulation of prostaglandin E2 has been linked to increased secretion of matrix metalloproteinases [37, 38], which ultimately could lead to the decomposition of collagen in cartilage and other ECM components that form the backbone of menisci. These observations are consistent with our finding of changes related to the metabolism or biosynthesis of glycolipids, arachidonic acids, and steroid hormones, although such alterations did not seem to exhibit statistical significance. Therefore, our data suggested that early ACL injury could also be linked to perturbations in steroid-based regulators of metabolism in synovial fluid, which could contribute to local cartilage and meniscal remodeling.

Glycerophospholipid metabolism in synovial fluid was found to be correlated with elevated T2 relaxation times of cartilage and meniscus in the current study. Since the elevated T2 relaxation times were confirmed to be related to the degeneration of cartilage and meniscus [7, 8], glycerophospholipid metabolism related compounds could be considered as potential biomarkers for the onset of posttraumatic osteoarthritis after early ACL injury. As metabolic changes in synovial fluid often precede detectable degenerative alterations in cartilage and meniscus, monitoring glycerophospholipid levels might serve as a useful strategy for capturing early pathophysiological signatures of cartilage and meniscus degeneration after ACL injury. The further mechanism research should be done to elucidate the exact role of glycerophospholipid on cartilage and meniscus.

There were several limitations in this study. First of all, our study had a limited sample size, which may affect the accuracy of the results. Secondly, we only focused on the onset (i.e., the first 4 weeks) of posttraumatic osteoarthritis after ACL injury and did not assess the longitudinal degenerative changes. This is based on previous evidence that degenerative changes could occur at 4-12 weeks, and moderate/severe OA at 8 weeks, after ACL-injury [17]. Future metabolomic studies could focus on the elucidation of metabolic changes along the progression of osteoarthritis after ACL injury and its correlation with biochemical changes of cartilage and meniscus. A larger sample size would also be essential to ensure the accuracy and reliability of the results.

## 5. Conclusions

In summary, ACL injury of rabbit knees resulted in elevated T2 relaxation times of cartilage and meniscus, and perturbed metabolism of various lipids and steroids, particularly glycerophospholipids. As glycerophospholipid metabolism related compounds were correlated with elevated T2 relaxation times of cartilage and meniscus, these compounds could be considered as potential biomarkers for early degenerative changes of cartilage and meniscus after ACL injury. Our results could facilitate the mechanistic elucidation of the onset of posttraumatic osteoarthritis after ACL injury.

## Figures and Tables

**Table 1:** Enriched metabolic pathways as determined by MetaboAnalyst 3.0 from the differential metabolites.

Name	Total	Expected	Hits	Raw *P*	Impact
Glycerophospholipid metabolism	30	0.21	3	0.00096	0.33
Primary bile acid biosynthesis	46	0.33	2	0.0401	0.01
Glycosylphosphatidylinositol(GPI)-anchor biosynthesis	14	0.10	1	0.0958	0.04
Glycerolipid metabolism	18	0.13	1	0.122	0.02
Arachidonic acid metabolism	36	0.26	1	0.23	0
Biosynthesis of unsaturated fatty acids	42	0.30	1	0.263	0
Steroid hormone biosynthesis	70	0.50	1	0.402	0.03

## Data Availability

The data used to support the findings of this study are included within the manuscript.
